# Health workers’ perception of digital technology use to improve mental health services

**DOI:** 10.4102/jphia.v16i1.1337

**Published:** 2025-06-13

**Authors:** Olubunmi Y. Fashoto, Maureen N. Sibiya, Olanrewaju Oladimeji

**Affiliations:** 1 Department of Health Sciences, Faculty of Health Sciences, Durban University of Technology, Durban, South Africa; 2 Department of Psychology, Faculty of Applied Social Sciences, Eswatini Medical Christian University, Mbabane, Eswatini; 3 Mangosuthu University of Technology, Durban, South Africa; 4 Department of Epidemiology and Biostatistics, School of Public Health, Sefako Makgatho Health Sciences University, Pretoria, South Africa

**Keywords:** digital technologies, healthcare workers, mental health, perception, well-being

## Abstract

**Background:**

Integrating digital technologies into mental healthcare offers a transformative shift in the provision of online-based mental health services to Nigerians. This is crucial for Nigeria in order to minimise and curb the developing health emergency because of COVID-19.

**Aim:**

This study seeks to explore the perception of healthcare workers (HCWs) towards the use of digital technologies in improving mental services in Nigeria.

**Setting:**

This study was carried out in four neuropsychiatric hospitals in South-West Nigeria.

**Methods:**

Mixed-method convergent design was used. Purpose sampling utilising snowball method was utilised in selecting qualitative participants, while proportional stratified sampling method was adopted for quantitative participants. Quantitative data collection tools were administered to 317 participants at the selected neuropsychiatric hospitals while interview was conducted on 16 qualitative participants. Thematic analysis was employed on qualitative data and descriptive statistical analysis (mean, standard deviation, frequencies, percentages, crosstabs, Chi-square) was conducted on quantitative data.

**Results:**

Findings showed that 58% of psychiatric nurses, 17% of clinical psychologists, 16% of psychiatrists and 6% of occupational therapists view digital technologies as effective in combination with face-to-face therapy. Additionally, qualitative findings showed that HCWs view digital tools as helpful tools for both patients and HCWs and offer the opportunity to immensely advance mental services.

**Conclusion:**

Incorporating digital technologies into mental health service delivery enhances mental health services, increases prompt access to healthcare and reduces the effect and impact of any future health emergencies.

**Contribution:**

This study raised awareness on the need to improve mental health services in public mental health facilities and advocate for the integration of digital technologies to improve mental health service delivery.

## Introduction

The mental health and well-being of the population should be a top priority in every society because mental health problems are contributory factors to global health problems and affect every facet of life physically, socially, emotionally, economically, educationally and psychologically, and also impact individuals about how they interact within their environment. In developed countries, digital technologies were adopted in various healthcare systems before the COVID-19 pandemic to meet up with growing needs. Digital tools led to the emergence of different online support systems such as ‘informational support’ and ‘network support’ in Taiwan.^1^ Findings from Sweden and Finland found some participants had unfavourable perception of digital health intervention because they were unable to use them because of their lack of skills, while other participants perceived them as smart and effective intervention as a result of their competence in using them.^2^ Several therapeutic interventions, combined with digitalised programmes aid in preventing suicide among the youth during the pandemic.^3,4^ Beyond the general population, mental health issues affect people with pre-existing conditions, drug addiction, people with alcoholism,^5^ first responders,^6^ and people at risk of the pandemic, such as the old, children and people living with chronic illnesses. Telemedicine was used during the Ebola outbreak and COVID-19 pandemic.^7^ In South Africa and some other African countries, telemedicine has been successfully utilised for ophthalmology using videoconferencing, teledermatology and pathology, and for continuous learning.^8^ Teleconsulting was employed in Uganda to provide mental health support during the pandemic; additionally, a virtual online programme was used in Kenya to reach people with mental health needs during the COVID-19 pandemic.^7^

The use of short message services (SMSs) as a reminder reduces missed appointments among patients newly diagnosed with mental illness in Nigeria.^9^ Few studies reported the use of telemedicine to provide different healthcare services in Nigeria during the COVID pandemic and those who utilised it faced poor network connectivity, inconsistent power supply and unavailability of needed equipment.^10,11^ Digital technologies enhance efficiency, increase performance and do not impede therapist–patient relationship, and it is viewed as an important component needed in healthcare system^12,13^; however, the utilisation is low.^14,15^ These findings are not uncommon, especially among the Nigerian population and several studies consistently found that perceptions about mental illness are caused by supernatural powers, evil spirits or witches, and hence the reluctance to utilise digital mental health services.^16,17^

Nigeria’s population is around 200 million, and the integration of digital health technology into mental healthcare can leverage care beyond in-person care at mental health facilities and easy accessibility to services.^18^ The effective integration of digital tools enhances the efficiency of mental healthcare and also aids in preparing for any futuristic infectious outbreaks or pandemics. This study seeks to explore the perception of healthcare workers (HCWs) towards the use of digital technologies to improve mental healthcare services in Nigeria.

## Research methods and design

### Study design and sampling

This study adopted a convergent mixed methods approach. Questionnaires assessing HCWs’ perception of using digital technologies for mental health services were distributed to selected participants in the four neuropsychiatric hospitals and interviews. Furthermore, interviews were conducted in the selected facilities. Proportional stratified sampling utilising Taro Yamane 1973 formula was used in selecting the participants for the quantitative study:^19^

$$n=N/(1+{\text{Ne}}^{2})$$ [Eqn 1]

where *n* = sample size, *N* = total population, and *e* = margin error value which is 0.05.

A 5% non-response rate was computed to increase the sample size from *n* = 317 to a total of 337. A total of 337 questionnaires were distributed; however, 17 questionnaires were not returned, while 3 questionnaires had incomplete information. Qualitative study employs purposive sampling method using the snowball method (*n* = 16) in which previously informed HCWs identify other HCWs for the interview. This study utilised snowball sampling technique because it increases the rate of respondent participation.^20^ Nigeria HCWs are very busy because of their hectic work schedule, and scheduling an appointment for an interview is challenging, hence, the use of snowball sampling to identify willing participants. Despite the pros of snowball sampling, it has its shortcomings. Snowball sampling leads to sampling bias, as the sample may not be the true representation of the population. Sampling bias makes it difficult to generalise findings from the study on the entire population. One-on-one interviews were conducted to collect data because of the sensitive nature of the questions and to generally ensure the accuracy of the data. Consent form was given to participants to sign before commencing the interview.

### Setting and population

The study was conducted in the south-west geo-political zone in Nigeria. The south-west geo-political zone comprises six states namely Ondo, Ogun, Osun, Lagos, Oyo and Ekiti State. Four government-owned psychiatric hospitals in the south-west geo-political zone were studied, namely, the Neuropsychiatric Hospital Yaba, Lagos State. The institution provides training for resident psychiatrists, and has both in-patient and out-patient treatment; Aro Neuropsychiatric Hospital Abeokuta, Ogun State. Aro is the first psychiatric hospital to debut a School of Psychiatric Nursing. It provides both in-patient and out-patient services and is invested in mental health research^21^; Department of Psychiatry, University of Ibadan Teaching Hospital, Oyo State. The Psychiatry unit of the hospital provides both theoretical and practical training for psychiatrists and creates a platform for research. It provides psychiatric care to both adults and children within the state and its environs^22^ and Neuropsychiatric Hospital, Akure, Ondo State provides mental health services to people within and outside the state.^23^ Healthcare workers with less than 2 years of working experience, students on internship, administrative staff and participants who did not give consent were excluded from the study.

### Data collection and analysis

The questionnaire used for quantitative data collection was divided into five sections: demographic characteristics, use of digital technology, HCWs’ specialisation and digital technologies used and perception of HCWs about the use of digital technologies and challenges to the provision of mental healthcare services.

Questionnaires were administered during office hours and retrieved after a few days, additionally, three research assistants were trained on the distribution, collection of questionnaires and adhere to ethical standard when handling participant responses. All three authors developed the semi-structured interview which provides robust insight and thoroughness and probes participants’ subjective views. It explored participants’ views on and perception of digital technologies, and challenges in utilising digital technologies for service delivery. Questions such as, ‘*What is your opinion about the level at which digital technology is being embraced for the provision of mental healthcare services?*’ were explored. Thus, all items on the scale were considered reliable to measure the variable of interest. Participant’s permission was sought before audio taping the interview. The IBM^®^ SPSS^®^, version 20 was employed to compute descriptive analysis (e.g., mean, standard deviation, frequencies, percentages, crosstabs, Chi-square) to provide an overall picture of quantitative data analysis. Thematic analysis was conducted on qualitative data. Participants’ responses were audio recorded and later transcribed and codes were generated. Themes were identified and presented. Findings from qualitative and quantitative analysis are presented and integrated to provide a broader picture of the study under investigation.

### Ethical considerations

Ethical approval was obtained from the Durban University of Technology Research and Ethics Committee, South Africa (IREC Reference number 164/22), and permission was approved by the Ethics Committees at the facilities used in the study. Consents were also obtained from participants.

## Results

### Demographic characteristics

Table 1 shows the demographic characteristics of quantitative participants which include age, gender, facility location and experience in years. Findings show Clinical psychologists and Psychiatric nurses have more participants within the 20–29-year age range (7% respectively). Majority of the participants are female (65%) while male participants are (34%). The data on gender revealed that majority of individuals who were surveyed identified as female, comprising 9% Clinical psychologists, 44% Psychiatric nurses and 8% Psychiatric doctors. Among male participants 7% were Clinical psychologists, 15% Psychiatric nurses and 7% Psychiatric doctors.

**TABLE 1:** Demographic characteristics of quantitative participants (*N* = 317).

Demographic characteristics	Clinical psychologist		Occupational therapist		Psychiatric doctor		Psychiatric nurse		Total	
	*n*	%	*n*	%	*n*	%	*n*	%	*n*	%
**Age group† (years) (*n* = 313)**										
20–29	22	7.1	3	1.0	6	1.9	22	7.1	53	17.1
30–39	22	7.1	9	2.9	29	9.4	83	26.8	143	46.1
40–49	8	2.6	4	1.3	14	4.5	58	18.7	84	27.1
Above 50	1	0.3	1	0.3	2	0.6	26	8.4	30	9.7
**Gender† (*n* = 294)**										
Female	27	9.3	10	3.4	25	8.6	129	44.3	191	65.6
Male	22	7.6	10	3.4	23	7.9	45	15.5	100	34.4
**Facility location (*n* = 317)**										
Akure	3	1.0	2	0.6	3	1.0	26	8.3	34	10.8
Abeokuta	19	6.1	13	4.1	18	5.7	68	21.7	118	37.6
Ibadan	9	2.9	4	1.3	13	4.1	43	13.7	69	22.0
Yaba	22	7.0	1	0.3	17	5.4	53	16.9	93	29.6
**Experience† (years) (*n* = 313)**										
2–5	36	11.6	7	2.3	23	7.4	64	20.6	130	41.9
6–10	7	2.3	6	1.9	16	5.2	29	9.4	58	18.7
11–15	2	0.6	4	1.3	8	2.6	30	9.7	44	14.2
16–20	5	1.6	2	0.6	1	0.3	27	8.7	35	11.3
21–25	1	0.3	1	0.3	2	0.6	23	7.4	27	8.7
26–30	0	0.0	0	0.0	1	0.3	11	3.5	12	3.9
31–35	0	0.0	0	0.0	0	0.0	2	0.6	2	0.6
Above 35	0	0.0	0	0.0	0	0.0	2	0.6	2	0.6

† , Variables with missing data.

Majority of the HCWs have work experience between 2 years and 25 years while most Psychiatric nurses recorded over 35 years of work experience. For each specialisation, 20.6% of Psychiatric nurses, 11.6% of Clinical psychologists, 7.4% of Psychiatrists and 2.3% of Occupational therapists have 2–5 years of work experience. Aro has the highest number of participants (37%) while Akure has the lowest number of participants (10%).

Qualitative participants are mostly female (*n* = 9) as compared to male participants (*n* = 7) as shown in Table 2. The age of participants for qualitative study ranges from 20 years to 29 years (3 participants), 30 years to 39 years (7 participants) and 40 years to 49 years (5 participants) while only 1 participant was above 50 years. The number of Psychiatric nurses (*n* = 5) who participated in the interview was higher when compared to other specialisations (Clinical psychologist = 4, Psychiatric doctor = 4 and Occupational therapist = 3). Majority of the participants (*n* = 10) have more than 11 years of working experience while 6 have less than 10 years of working experience in mental healthcare.

**TABLE 2:** Demographic characteristics of qualitative participants.

Participants	Value
**Gender**	
Male	7
Female	9
**Age (years)**	
20–29	3
30–39	7
40–49	5
Above 50	1
**Area of specialisation**	
Psychiatric nurse	5
Clinical psychologist	4
Occupational therapist	3
Psychiatric doctor	4
**Years in experience**	
2–5	3
6–10	3
11–15	5
16–20	4
Above 20	1

All who participated in the interview have used digital technologies before and during COVID-19 to provide mental health services such as intervention, therapy, appointment booking, consultation, follow-up and prescription of medications. Code for qualitative participants includes:

- Neuropsychiatric hospital Yaba: #YAP1,.., #YAP4.
- Aro Neuropsychiatric hospital: #ARP1,.. #ARP4.
- Department of Psychiatry, University of Ibadan: #IBP1,…#IBP4.
- Neuropsychiatric Hospital, Akure: #AKP1, #AKP2,…#AKP4.

Two main themes and five sub-themes emanated from the qualitative findings such as perception of digital tools (sub-themes: attitude towards digital tools, acceptability, patient care) and challenges affecting the utilisation of digital technologies for mental health service delivery (sub-themes: operational issues, policy and perceived extra burden on HCWs).

### Use of digital technologies

Despite having adequate knowledge about these digital tools, their use is minimal. Only 137 (43.2%) HCWs indicated that they used digital tools in their day-to-day provision of services such as consulting, prevention and treatment while 178 (56.2%) said they did not use digital technologies in their day-to-day provision of services as presented in Table 3. Qualitative findings resonate that HCWs have adequate knowledge of digital technologies. Healthcare workers are aware of WhatsApp both for calls and chat, Zoom, telephone, Google Meet, Electronic Medical Record System, Telemedicine and media platforms to mention a few. Findings further highlight that symptoms of anxiety can be effortlessly hidden or disguised on virtual platforms. Emotional changes are hard to identify on digital technologies, which is the reason some HCWs are resisting the incorporation of digital technologies into mental health services. This is noted in a study that physicians have challenges utilising digital tools to assess and evaluate patient’s physical state such as decreased weight or dermatological issues.^24^ This is further reinforced that it is impossible to conduct some treatment over digital platform.^25^ Similarly, physical contact is viewed as pertinent when it comes to care which digital technologies do not provide, fostering resistance to the use of digital technologies.^26^

**TABLE 3:** Use of digital technology with clients.

Use of digital technology	Yes		No	
	*n*	%	*n*	%
Use digital technology with clients	137	43.2	178	56.2

### Specialisation and types of digital technologies used

Table 4 presents digital technologies used by HCWs during the COVID pandemic ranging from Google Meet (9.1%), Telemedicine (5.7%) and WhatsApp call (8.8%), to Zoom (6.3%); however, Skype, multimedia and telegraphy had low usage (< 0.3%). Clinical psychologists utilised Zoom calls more (50%) as compared to Occupational therapists (5%), Psychiatrists (15%) and Psychiatric nurses (30%). Psychiatric nurses (53.6%) as compared to Clinical psychologists (21.4%) used telephone calls more while Occupational therapists rarely made phone calls. Clinical psychologists and Occupational therapists did not use telemedicine but Psychiatrists and Psychiatric nurses adopted telemedicine for service delivery. It is interesting to note that Psychiatric nurses are the only HCWs who adopted Skype during the COVID pandemic. Findings also indicated that about 20% of Psychiatric doctors and 4% of Psychiatric nurses significantly used telemedicine for consultation, evaluation and treatment during the pandemic. There is a relationship between HCWs’ specialisation and the use of Zoom (*p* < 0.05), telemedicine (*p* < 0.05), WhatsApp (*p* < 0.05) and Google Meet (*p* < 0.05). Clinical psychologists use Zoom (3%), WhatsApp (4%) and Google Meet (5%) more than other HCWs. Similarly, Psychiatric doctors (3%) utilise telemedicine more when compared to other HCWs.

**TABLE 4:** Healthcare workers’ specialisation and type of digital technologies used.

Digital technologies tools	Specialisation								Total (*n* = 314)		*P*
	Clinical psychologist (*n* = 53)		Occupational therapist (*n* = 20)		Psychiatric doctor (*n* = 51)		Psychiatric nurse (*n* = 190)				
	*n*	%	*n*	%	*n*	%	*n*	%	*n*	%	
**Zoom**	-	-	-	-	-	-	-	-	-	-	0.002
Yes	10	3.2	1	0.3	3	1.0	6	1.9	20	6.4	-
**Telemedicine**	-	-	-	-	-	-	-	-	-	-	< 0.001
Yes	0	0.0	0	0.0	10	3.2	8	2.5	18	5.7	-
**Skype**	-	-	-	-	-	-	-	-	-	-	0.292
Yes	1	0.3	0	0.0	0	0.0	0	0.0	1	0.3	-
**Calls**	-	-	-	-	-	-	-	-	-	-	0.387
Yes	6	1.9	0	0.0	7	2.2	15	4.8	28	8.9	-
**WhatsApp**	-	-	-	-	-	-	-	-	-	-	0.003
Yes	11	3.5	0	0.0	7	2.2	10	3.2	28	8.9	-
**Google Meet**	-	-	-	-	-	-	-	-	-	-	< 0.001
Yes	16	5.1	2	0.6	6	1.9	5	1.6	29	9.2	-
**Telegraphy session**	-	-	-	-	-	-	-	-	-	-	0.981
Yes	1	0.3	0	0.0	1	0.3	3	1.0	5	1.6	-
**Multimedia**	-	-	-	-	-	-	-	-	-	-	0.956
Yes	0	0.0	0	0.0	0	0.0	1	0.3	1	0.3	-

### Perceptions of healthcare workers towards the utilisation of digital technologies

Table 5 highlights that about 16% of Psychiatric doctors, 14% of Clinical psychologists, 5% of Occupational therapists and 53% of Psychiatric nurses perceived that digital technologies would have a positive impact on client’s mental health. Likewise, approximately 58% of Psychiatric nurses, 17% of Clinical psychologists, 16% of Psychiatrists and 6% of Occupational therapists view digital technologies as effective in combination with face-to-face therapy. Approximately 54% of Psychiatric nurses, 14% of Clinical psychologists, 16% of Psychiatrists and 5% of Occupational therapists view digital technologies as beneficial. About 13% of Clinical psychologists, 50% of Psychiatric nurses, 12% of Psychiatrists and 5% of Occupational therapists view that digital technologies provide more data security with patient information while 16% of Psychiatric doctors, 14% of Clinical psychologists, 52% of Psychiatric nurses and 5% Occupational therapists opined that digital technologies for mental health intervention will be credible.

**TABLE 5:** Perception of healthcare workers about the use of digital technologies.

Variables	Specialisation								Total (*n* = 314)		*P*
	Clinical psychologist (*n* = 53)		Occupational therapist (*n* = 20)		Psychiatric doctor (*n* = 51)		Psychiatric nurse (*n* = 190)				
	*n*	%	*n*	%	*n*	%	*n*	%	*n*	%	
**Perception of HCWs about the use of digital technologies:**											
The impact of digital technologies on clients’ mental health will be positive.	-	-	-	-	-	-	-	-	-	-	0.015
Yes	44	14.0	14	4.5	50	15.9	173	55.1	281	89.5	-
No	9	2.9	6	1.9	1	0.3	17	5.4	33	10.5	-
Digital technologies for providing mental healthcare services will do more good to clients.	-	-	-	-	-	-	-	-	-	-	0.020
Yes	44	14.0	15	4.8	51	16.2	158	50.3	268	85.4	-
No	9	2.9	5	1.6	0	0.0	32	10.2	46	14.6	-
The use of digital technologies to provide mental healthcare services is pleasant.	-	-	-	-	-	-	-	-	-	-	0.075
Yes	47	15.0	17	5.4	51	16.2	161	51.3	276	87.9	-
No	6	1.9	3	1.0	0	0.0	29	9.2	38	12.1	-
The use of digital technologies for clients’ mental health will be highly beneficial.	-	-	-	-	-	-	-	-	-	-	0.005
Yes	45	14.3	14	4.5	51	16.2	171	54.5	281	89.5	-
No	8	2.5	6	1.9	0	0.0	19	6.1	33	10.5	-
Digital interventions for clients’ mental health will be credible.	-	-	-	-	-	-	-	-	-	-	0.053
Yes	45	14.3	16	5.1	51	16.2	164	52.2	276	87.9	-
No	8	2.5	4	1.3	0	0.0	26	8.3	38	12.1	-
When using digital technologies, the personal information provided by clients is mostly secured.	-	-	-	-	-	-	-	-	-	-	0.440
Yes	40	12.7	15	4.8	38	12.1	158	50.3	251	79.9	-
No	13	4.1	5	1.6	13	4.1	32	10.2	63	20.1	-
Regardless of the severity of symptoms, digital interventions are typically effective.	-	-	-	-	-	-	-	-	-	-	0.199
Yes	15	4.8	7	2.2	14	4.5	41	13.1	77	24.5	-
No	38	12.1	13	4.1	37	11.8	149	47.5	237	75.5	-
A strong working alliance with clients is achievable when using digital technologies to provide mental healthcare services.	-	-	-	-	-	-	-	-	-	-	0.625
Yes	38	12.1	12	3.8	37	11.8	119	37.9	206	65.6	-
No	15	4.8	8	2.5	14	4.5	71	22.6	108	34.4	-
When digital interventions are combined with face-to-face therapies, they can be more effective.	-	-	-	-	-	-	-	-	-	-	0.945
Yes	52	16.6	19	6.1	50	15.9	183	58.3	304	96.8	-
No	1	0.3	1	0.3	1	0.3	7	2.2	10	3.2	-
**Attitude of HCWs towards the use of digital technologies:**											
A mental health professional should have complete control over whether to use digital technologies with clients or not.	-	-	-	-	-	-	-	-	-	-	0.016
Yes	35	11.1	16	5.1	43	13.7	158	50.3	252	80.3	-
No	18	5.7	4	1.3	8	2.5	32	10.2	62	19.7	-
There is confidence generally in the ability of mental health professionals to use digital technologies with clients.	-	-	-	-	-	-	-	-	-	-	0.912
Yes	30	9.6	12	3.8	28	8.9	105	33.4	175	55.7	-
No	23	7.3	8	2.5	23	7.3	85	27.1	139	44.3	-
It is mostly up to mental healthcare professionals whether they decide to use digital technologies with clients or not.	-	-	-	-	-	-	-	-	-	-	0.008
Yes	41	13.1	18	5.7	26	8.3	127	40.4	212	67.5	-
No	12	3.8	2	0.6	25	8.0	63	20.1	102	32.5	-
Most mental healthcare professionals can use digital technologies to provide mental healthcare services for clients.	-	-	-	-	-	-	-	-	-	-	< 0.001
Yes	25	8.0	13	4.1	35	11.1	151	48.1	224	71.3	-
No	28	8.9	7	2.2	16	5.1	39	12.4	90	28.7	-
A mental health professional should be confident in their ability to use digital technologies to deliver mental health interventions to clients.	-	-	-	-	-	-	-	-	-	-	0.001
Yes	42	13.4	18	5.7	50	15.9	181	57.6	291	92.7	-
No	11	3.5	2	0.6	1	0.3	9	2.9	23	7.3	-
The required technical knowledge to use digital interventions for clients’ mental healthcare needs is necessary.	-	-	-	-	-	-	-	-	-	-	< 0.001
Yes	43	13.7	14	4.5	51	16.2	177	56.4	285	90.8	-
No	10	3.2	6	1.9	0	0.0	13	4.1	29	9.2	-
Most mental healthcare professionals across other geographies are using digital technology effectively with their clients.	-	-	-	-	-	-	-	-	-	-	0.001
Yes	46	14.6	11	3.5	49	15.6	149	47.5	255	81.2	-
No	7	2.2	9	2.9	2	0.6	41	13.1	59	18.8	-
**Acceptability of the use of digital technologies among HCWs:**											
Most mental healthcare professionals would support the use of digital technology with clients.	-	-	-	-	-	-	-	-	-	-	< 0.001
Yes	46	14.6	10	3.2	51	16.2	147	46.8	254	80.9	-
No	7	2.2	10	3.2	0	0.0	43	13.7	60	19.1	-
It is important for ‘mental health professional bodies’ to support the use of digital technology within practice.	-	-	-	-	-	-	-	-	-	-	0.062
Yes	47	15.0	17	5.4	51	16.2	179	57.0	294	93.6	-
No	6	1.9	3	1	0	0.0	11	3.5	20	6.4	-
Most mental healthcare professionals would approve of the use of digital interventions with clients.	-	-	-	-	-	-	-	-	-	-	< 0.001
Yes	44	14.0	10	3.2	50	15.9	166	52.9	270	86.0	-
No	9	2.9	10	3.2	1	0.3	24	7.6	44	14.0	-
Most mental healthcare professionals would support the use of digital technology in therapy.	-	-	-	-	-	-	-	-	-	-	< 0.001
Yes	47	15.0	9	2.9	50	15.9	155	49.4	261	83.1	-
No	6	1.9	11	3.5	1	0.3	35	11.1	53	16.9	-
Digital interventions are effective in treating mental health concerns.	-	-	-	-	-	-	-	-	-	-	< 0.001
Yes	47	15.0	12	3.8	49	15.6	142	45.6	250	79.6	-
No	6	1.9	8	2.5	2	0.6	48	15.3	64	20.4	-
Compared to face-to-face therapy, digital technologies are equally effective in treating mental health concerns.	-	-	-	-	-	-	-	-	-	-	0.002
Yes	37	11.8	7	2.2	42	13.4	122	38.9	208	66.2	-
No	16	5.1	13	4.1	9	2.9	68	21.7	106	33.8	-
**Patient care:**											
The use of digital interventions with clients should be encouraged to continue.	-	-	-	-	-	-	-	-	-	-	0.005
Yes	48	15.3	14	4.5	51	16.2	159	50.6	272	86.6	-
No	5	1.6	6	1.9	0	0.0	31	9.9	42	13.4	-
It is important to have a plan to use digital technology for mental healthcare services with clients.	-	-	-	-	-	-	-	-	-	-	0.007
Yes	46	14.6	14	4.5	51	16.2	165	52.5	276	87.9	-
No	7	2.2	6	11.9	0	0.0	25	8.0	38	11.2	-
It is ideal to use digital technology with clients when face-to-face therapy is not feasible.	-	-	-	-	-	-	-	-	-	-	0.005
Yes	51	16.2	15	4.8	51	16.2	172	54.8	289	92	-
No	2	0.6	5	1.6	0	0.0	18	5.7	25	8.0	-
It is becoming more likely that the use of digital interventions for clients’ mental healthcare will be inevitable.	-	-	-	-	-	-	-	-	-	-	0.001
Yes	52	16.6	17	5.4	50	15.9	150	47.8	269	85.7	-
No	1	0.3	3	1.0	1	0.3	40	12.7	45	88.9	-
Having the necessary resources to use digital technology with clients is important.	-	-	-	-	-	-	-	-	-	-	0.051
Yes	52	16.6	17	5.4	51	16.2	178	56.7	298	94.9	-
No	1	0.3	3	1.0	0	0.0	12	3.8	16	5.1	-
Learning more about digital technologies in mental healthcare service provision is important.	-	-	-	-	-	-	-	-	-	-	0.118
Yes	53	16.9	18	5.7	51	16.2	179	57	301	95.9	-
No	0	0.0	2	0.6	0	0.0	11	3.5	13	4.1	-
It is important to make time to learn about digital interventions for mental healthcare services.	-	-	-	-	-	-	-	-	-	-	0.208
Yes	50	15.9	18	5.7	51	16.2	171	54.5	290	92.4	-
No	3	1.0	2	0.6	0	0.0	19	6.1	24	7.6	-
Clients will like to use digital interventions to address their mental health concerns.	-	-	-	-	-	-	-	-	-	-	< 0.001
Yes	51	16.2	14	4.5	49	15.6	142	45.2	256	81.5	-
No	2	0.6	6	1.9	2	0.6	48	15.3	58	18.5	-

HCW, healthcare workers.

This study also found a relationship between HCWs’ perception of digital technologies and the use of digital technologies in the provision of mental health services (*p* < 0.05). Most HCWs perceived digital tools to be reliable to an extent while few perceived them as unreliable in the qualitative findings. The dangers of missing important facial and bodily cues during sessions are the major highlights with the non-usage of digital tools in the qualitative findings which impact their perception of digital technologies.

### Attitude towards digital technologies

Table 5 revealed that approximately 10% of Clinical psychologists, 9% of Psychiatric doctors, 4% of Occupational therapists and 33% of Psychiatric nurses have confidence in HCWs’ capacity to use digital technologies in providing services, while 7% of Clinical psychologists, 3% of Occupational therapists, 7% of Psychiatric doctors and 27% of Psychiatric nurses do not have confidence in the use of digital technologies for service delivery. About 13% of Clinical psychologists, 6% of Occupational therapists and 8% of Psychiatric doctors indicated that healthcare professionals would support the utilisation of digital technologies with clients. However, reports from qualitative analysis showed that most HCWs had positive attitude towards the use of digital technologies, and some were not open to the use of digital tools in service delivery. Some HCWs relied heavily on the traditional method of service delivery because of the possibility of missing important facial or bodily cues on digital technologies.

Furthermore, in the qualitative findings, quite a number of HCWs were open to the use of digital technologies with their patients while some were totally against the utilisation of digital technologies for mental health service delivery. Healthcare workers quoted the inability to view bodily cues (e.g., leg shaking, palm sweating, etc.) and patients’ camouflaging of their emotions, as some of the reasons why the use of digital technologies would be disadvantageous in service delivery. Factors such as reliance on the manual way of doing things, lack of awareness or education on digital health and lack of incentives are possible reasons for HCWs’ resistance.

### Acceptability of the use of digital technologies

Approximately 2% of Occupational therapists, 12% of Clinical psychologists, 13% of Psychiatric doctors and 40% of Psychiatric nurses highlight that digital technologies are as effective as face-to-face intervention in treating mental health concerns as shown in Table 5. This means that the acceptability of digital tools by HCWs is high. Furthermore, about 15% of Clinical psychologists, 16% of Psychiatric doctors, 3% of Occupational therapists and 47% of Psychiatric nurses opined that mental health professional bodies should support and approve the use of digital intervention with clients. Similarly, about 4% of Occupational therapists and 22% of Psychiatric nurses still believe that the use of digital technologies cannot be as effective as face-to-face intervention. Qualitative findings support results from quantitative data on the acceptability of digital technologies as they improve the therapy process and increase prompt access to mental health services. As in-person meetings are reduced during the pandemic, they can have sessions with their patients in their comfort zones.

### Patient care

Table 5 shows that about 16% of Psychiatric doctors, 16% of Clinical psychologists, 5% of Occupational therapists and 45% of Psychiatric nurses noted that clients would like to use digital intervention for their mental health needs. Similarly, approximately 5% of Occupational therapists, 16% of Psychiatric doctors, 17% of Clinical psychologists and 57% of Psychiatric nurses concur that having the necessary resources to use digital tools with clients is important. Approximately 6% of Occupational therapists, 16% of Psychiatric doctors, 17% of Clinical psychologists and 57% of Psychiatric nurses believe that there is need for continuous learning about digital technologies in the provision of mental health services. About 5% of Occupational therapists, 55% of Psychiatric nurses, 16% of Clinical psychologists and Psychiatric doctors view digital technologies allows consultation, follow-up, therapy or treatment that can continue without restrictions. Invariably, if there is another lockdown or restriction of movement digital platform can be utilised. Qualitative findings showed that, in as much as digital technologies are beneficial to both patients and HCWs, some are of the view that it is inadequate. Incorporating these tools into daily care would make it easier to reach patients to ensure that their treatments are not derailed but there is still need for caution.

### Challenges affecting the utilisation of digital technologies for mental health

#### Operational issues

Some operational issues came up from participants such as navigation problems because of several options on the application (such as sharing screens on Zoom for individual or group therapy) (mean = 3.4, standard deviation [s.d.] = 1.2). Patients are responsible for financial implication incurred during online therapy sessions, and sometimes HCWs incur subscription costs in order to check on their patients. High data consumption is another issue noted by HCWs (mean = 3.5, s.d. = 0.1). Some HCWs differ on the view of inefficiency of the current digital tools in mental health facilities (mean = 2.3, s.d. = 0.9). Likewise, some participants have a challenge with high data subscription rates (mean = 3.4, s.d. = 1.0) as shown in Table 6. Findings in the interview pointed out that Nigeria is a country with constant power cuts, which hinders the smooth running of Wi-Fi. Inconsistent electricity supply is also another major barrier to effective digital intervention. Psychiatric hospitals need stable power supply to function. Poor network connectivity is a concern when discussing the barriers to the provision of mental health services through digital technologies in Nigeria.

**TABLE 6:** Challenges to the provision of mental healthcare services.

Variables	Specialisation								Total (*n* = 314)		Mean	s.d.
	Clinical psychologist (*n* = 53)		Occupational therapist (*n* = 20)		Psychiatric doctor (*n* = 51)		Psychiatric nurse (*n* = 190)					
	*n*	%	*n*	%	*n*	%	*n*	%	*n*	%		
**Digital technology for mental healthcare services usually has navigation problems because of having too many options**	-	-	-	-	-	-	-	-	-	-	3.4	1.2
Agree/strongly agree/moderately agree	38	12.1	14	4.5	40	12.7	122	38.9	214	68.4	-	-
Disagree/strongly disagree	15	4.8	6	1.9	11	3.5	67	21.3	99	31.6	-	-
A major challenge with digital technology for mental healthcare services is that they are quite complicated to use.	-	-	-	-	-	-	-	-	-	-	2.7	1.1
Agree/strongly agree/moderately agree	27	8.6	9	2.9	24	7.6	83	26.4	143	46.3	-	-
Disagree/strongly disagree	25	8.0	11	3.5	27	8.6	103	32.8	166	53.7	-	-
Digital technologies for mental healthcare services are not efficient.	-	-	-	-	-	-	-	-	-	-	2.3	0.9
Agree/strongly agree/moderately agree	18	5.7	10	3.2	10	3.2	47	15.0	85	27.3	-	-
Disagree/strongly disagree	35	11.1	10	3.2	41	13.1	140	44.6	226	72.7	-	-
Subscription cost for digital technology is very high and unaffordable for an average client.	-	-	-	-	-	-	-	-	-	-	3.4	1.0
Agree/strongly agree/moderately agree	35	11.1	16	5.1	45	14.3	151	48.1	247	79.7	-	-
Disagree/strongly disagree	18	5.7	4	1.3	5	1.6	36	11.5	63	20.3	-	-
Digital technologies for mental healthcare services involve heavy data usage, and that might be very expensive for an average client.	-	-	-	-	-	-	-	-	-	-	3.5	1.0
Agree/strongly agree/moderately agree	47	15.0	17	5.4	48	15.3	150	47.8	262	84.5	-	-
Disagree/strongly disagree	6	1.9	3	1.0	2	0.6	37	11.8	48	15.5	-	-
My organisation’s rules are against the utilisation of digital technologies for the provision of mental healthcare services and well-being.	-	-	-	-	-	-	-	-	-	-	2.1	1.0
Agree/strongly agree/moderately agree	15	4.8	11	3.5	6	1.9	52	16.6	84	27.0	-	-
Disagree/strongly disagree	38	12.1	9	2.9	44	14.0	136	43.3	227	73.0	-	-
My professional association is against the utilisation of digital technology for the provision of mental health services.	-	-	-	-	-	-	-	-	-	-	2.1	1.0
Agree/strongly agree/moderately agree	15	4.8	8	2.5	5	1.6	48	15.3	76	24.4	-	-
Disagree/strongly disagree	38	12.1	12	3.8	46	14.6	140	44.6	236	75.6	-	-
**Factors affecting the provision of mental healthcare services**												
Inadequate training and capacity development for mental health workers.	-	-	-	-	-	-	-	-	-	-	3.1	0.7
Little challenge/Challenge/serious challenge	53	16.9	19	6.1	51	16.2	178	56.7	301	96.2	-	-
Not a challenge	0	0.0	1	0.3	0	0.0	11	3.5	12	3.8	-	-
Poor/inappropriate mental healthcare policies by government.	-	-	-	-	-	-	-	-	-	-	3.3	0.7
Little challenge/Challenge/serious challenge	53	16.9	19	6.1	51	16.2	182	58	305	97.4	-	-
Not a challenge	0	0.0	1	0.3	0	0.0	7	2.2	8	2.5	-	-
Poor implementation/management of mental healthcare policies/programmes.	-	-	-	-	-	-	-	-	-	-	3.3	0.7
Little challenge/Challenge/serious challenge	53	16.9	20	6.4	51	16.2	186	59.2	310	99	-	-
Not a challenge	0	0.0	0	0.0	0	0.0	3	1.0	3	1.0	-	-
Lack of political will and motivation for mental health workers.	-	-	-	-	-	-	-	-	-	-	3.3	0.7
Little challenge/Challenge/serious challenge	52	16.6	20	6.4	51	16.2	186	59.2	309	98.7	-	-
Not a challenge	1	0.3	0	0.0	0	0.0	3	1.0	4	1.3	-	-

#### Policy

Healthcare workers had concerns with regulation and policy implementations so as to protect themselves. According to qualitative findings, the integration of digital technologies is not wrong, but regulatory framework that safeguards HCWs and patients needs to be implemented. Some of the HCWs utilising phones and social media for therapy sessions with patients are doing it at their own discretion while assuming full responsibility. The quantitative analysis result presented in Table 6 shows that HCWs concur that there are poor and inappropriate mental healthcare policies (mean = 3.3, s.d. = 0.7). Healthcare workers highlighted the lack of implementation of mental health policies (mean = 3.4, s.d. = 0.7) by the government, which is a barrier to a better mental health service provision in Nigeria.

However, despite the lack of policy and guidelines, some professional bodies as indicated by some HCWs (12% of Clinical psychologists, 4% of Occupational therapists, 15% of Psychiatric doctors and 45% of Psychiatric nurses) do not necessarily prevent HCWs from using digital technologies.

### Perceived extra burden on healthcare workers

Table 6 shows HCWs’ view that digital technologies are not complicated to use (mean = 2.7, s.d. = 1.1). Although HCWs echoed more on the lack of motivation for health professionals (mean = 3.3, s.d. = 0.7) and poor training and capacity development (mean = 3.1, s.d. = 0.7). There is inadequate knowledge on how to use some digital applications and extra burdens to HCWs which could jeopardise the use of digital technologies in mental healthcare.

The qualitative analysis extensively covered the perceived burden of the use of technologies on HCWs. Healthcare workers need to shift from their comfort zone and engage in rigorous training to use digital technologies efficiently and effectively. Some viewed the extra effort needed to get acquainted with digital technologies as an extra burden while some felt it is a way of self-development since the world is fast becoming digitalised.

## Discussion

The integration of digital technologies into mental healthcare will bridge the gap in service delivery.^27^ The advancement in digital technologies in the past decade aid the appropriateness, workability and benefits for mental healthcare interventions. Digital health technologies have been utilised to support mental health services; however, the COVID-19 pandemic provided a unique space for more use and evaluation of the efficacy of these digital health technologies.^28^ Various digital tools came in handy and were utilised by healthcare professionals in providing services in many countries during the lockdown stage and for contact tracing during the early days of the COVID-19 pandemic when contact tracing and isolation were the most reliable spread prevention methods, pending approved vaccines.^29^ The practice of using digital health technologies to provide mental health services has been generally abysmal.

Further, illiteracy and poor infrastructure including electricity supply and internet services, in addition to financial implication hamper the implementation of telemedicine and eHealth in Nigeria.^30^ Stable electricity supply has an economic implication for every society. The government needs to increase power generation to facilitate smooth operation in the mental healthcare sector and other sectors in the country. Furthermore, the Nigerian Government through the Ministry of Science, Information and Communication Technology, needs to collaborate with network service providers to provide sustainable and subsidised internet services and efficient technology devices with adequate data protection features to enhance mental healthcare service delivery and ensure preparedness for health emergencies. Moreover, Nigeria’s Government needs to increase the mental health sector budget to enable the purchase of technological devices to foster service delivery.

This study emphasised the need for constant training of HCWs on emerging technologies in order to utilise them effectively in service delivery. It is also noted that management needs to constantly train clinicians on technological gadgets to aid their adeptness in telemedicine.^18,31^ There is an urgent need for training and re-training of HCWs on the proper use of the available digital technologies and applications so as to facilitate easy and smooth integration of digital technologies into mental health service delivery. Thus, the human resource (HR) department in the public health sector should provide workshops and seminal presentations on knowledge and the use of digital technologies in mental healthcare service delivery. There should be continuous and adequate training, which will improve the usage of digital technologies for mental healthcare service delivery.

Factors such as reliance on the manual way of doing things, lack of awareness or education on digital health and lack of incentives are also other possible reasons for HCWs’ resistance. African countries face some emerging issues such as developing a legislative framework to safeguard mental health in the population and enhancing quality assurance to ensure the effective implementation of policies. The policy should guide both HCWs and patients on the level of the use of digital technology so as to prevent privacy infringement. Likewise, the healthcare sector is visibly underfunded, lacks appropriate regulations and is plagued with non-implementation of these policies because of the lack of political will and/or interest.^32^ This is further revealed in the lack of policies in the mental health sector which is evident in the *Lunacy Act* revisited in 1958 (allows HCWs and magistrates to confine mentally unstable individuals).^33^ However, the Act has undergone many amendments between 2003 and 2013 but the Nigerian Government has not signed the Act into law. More so, the 2019 Mental and Substance Abuse Bill aimed to strengthen the budget allocated to mental healthcare sector and improve services still remained unsigned.^33^ There is an urgent need for the Government to put an effective policy and framework in place to encourage the utilisation of digital technologies among HCWs.

The World Health Organization stipulated that by 2020, most countries would have launched or revised their mental health acts in accordance with the international human rights instrument set by the WHO Mental Health Action Plan 2013–2020.^33^ Additionally, obsolete mental health laws need to be reformed for effectiveness in service delivery, and new policies must be created to protect and support Nigerians who require mental health services.^27^ This study emphasised the need for constant training of HCWs on emerging technologies in order to utilise them effectively in service delivery. Hospital management needs to constantly train clinicians on technological gadgets to aid their adeptness in telemedicine.

The benefits of digital health significantly outweigh its shortcomings;^34^ thus, research interest in digital health has assumed an upsurge in recent times, and this has been further encouraged by the recent COVID-19 pandemic. The transitioning from conventional methods to an online-base system of mental health service delivery may increase the vulnerability of patient information to hackers; however, Information Technology expert (IT) can provide solution centred strategies. These strategies can include the use of pseudo names to represent each patient or the use of unique numbers as patient identifiers on the system.

### Implication for policymaker

This study emphasised the need for the Nigerian Government to develop strategies that will improve current mental healthcare services and evaluate facility-specific needs in the integration of digital technologies. The government should formulate policies that specify the extent and boundaries of use and also, incorporate the legal framework needed to protect HCWs and patient information and data privacy. Additionally, required finance for the successful integration of digital technologies needs to be highlighted in the policy. There is need for awareness among HCWs and the community on the different technological tools available for service delivery and the sensitisation on enormous benefits of digital technologies. Constant and continuous training should be embedded into psychiatric hospital staff development programmes so as to foster skill development and knowledge in using digital technologies.

## Conclusion

Digital technologies provide an opportunity to transform Nigeria’s mental health system by easing the burden on the scarce HCWs. The development and adoption of digital technologies provide leverage in the advancement of care; however, government, Hospital management, HCWs and patients determine the successful and continuous use of digital technologies. There is an urgent need for the government to put an effective policy and framework in place to encourage the utilisation of digital technologies among HCWs. Furthermore, the Nigerian Government through the Ministry of Health and Ministry of Information and Communication Technology, need to collaborate with Network Service providers to provide sustainable and subsidised internet services and efficient technology devices with adequate data protection features to enhance mental healthcare service delivery and ensure preparedness for health emergencies.
